# INTIMATE PARTNER VIOLENCE AMONG US OLDER IMMIGRANTS: WHAT ARE THE ODDS?

**DOI:** 10.1093/geroni/igae098.1403

**Published:** 2024-12-31

**Authors:** Rujeko Machinga-Asaolu, Yanghyun Park, Kathryn Showalter

**Affiliations:** University of Kentucky, Lexington, Kentucky, United States; University of Michigan, Ann Arbor, Michigan, United States; University of Kentucky, Lexington, Kentucky, United States

## Abstract

**Background:**

Intimate partner violence (IPV), a subtype of domestic violence, is a pervasive global problem with life-long impacts. Among U.S. older adults, the prevalence of psychological IPV (PAIPV) is 2.1%, and physical IPV (PVIPV) is 0.8%. Sadly, underrepresented individuals, including immigrants, have less access to resources that can serve as IPV protective factors. Due to the growing immigrant and older population in the U.S., it is essential to identify their unique IPV characteristics. Objective and Method: This study used and analyzed the latest publicly available cross-sectional data from the National Intimate Partner and Sexual Violence Survey 2016/17 (n=10,957) of a selected national sample of English and Spanish-speaking immigrants (n=1,121) to identify their odds of experiencing PAIPV or PVIPV-based on identified demographic characteristics, including age. Additionally, of interest and identified was the prevalence of PAIPV and PVIPV among older immigrants 65+ years (n=124).

**Results:**

Contrary to the literature, among older immigrants aged 65+ years, PVIPV (10.7%) was more prevalent than PAIPV (9.1%). In an adjusted regression model for PAIPV at 95% CI, Spanish and/or English-speaking immigrants aged 65+ years were 3.147 [1.065, 9.299] significantly more likely to experience PAIPV than younger immigrants. However, age was not significantly associated with PVIPV. To conclude, IPV presents itself in unique etiologies among older immigrants. This study is an initial step toward using national data to understand IPV better for a combined male and female sample of older immigrants in the U.S. Further discussed will be the distinct differences in the study findings.

